# A case–control study on pre-, peri-, and neonatal risk factors associated with autism spectrum disorder among Armenian children

**DOI:** 10.1038/s41598-024-63240-3

**Published:** 2024-05-29

**Authors:** Meri Mkhitaryan, Tamara Avetisyan, Anna Mkhoyan, Larisa Avetisyan, Konstantin Yenkoyan

**Affiliations:** 1 Neuroscience Laboratory, Cobrain Center, Yerevan State Medical University Named After M. Heratsi, 0025 Yerevan, Armenia; 2 Cobrain Center, Yerevan State Medical University Named After M. Heratsi, 0025 Yerevan, Armenia; 3 Muratsan University Hospital Complex, Yerevan State Medical University Named After M. Heratsi, 0075 Yerevan, Armenia; 4 Department of Infectious Diseases, Yerevan State Medical University Named After M. Heratsi, 0025 Yerevan, Armenia; 5 Department of Hygiene, Yerevan State Medical University Named After M. Heratsi, 0025 Yerevan, Armenia

**Keywords:** Autism spectrum disorder, Case–control study, Pregnancy, Risk factors, Autism spectrum disorders, Public health, Risk factors

## Abstract

We aimed to investigate the role of pre-, peri- and neonatal risk factors in the development of autism spectrum disorder (ASD) among Armenian children with the goal of detecting and addressing modifiable risk factors to reduce ASD incidence. For this purpose a retrospective case–control study using a random proportional sample of Armenian children with ASD to assess associations between various factors and ASD was conducted. The study was approved by the local ethical committee, and parental written consent was obtained. A total of 168 children with ASD and 329 controls were included in the analysis. Multivariable logistic regression analysis revealed that male gender, maternal weight gain, use of MgB6, self-reported stress during the pregnancy, pregnancy with complications, as well as use of labor-inducing drugs were associated with a significant increase in the odds of ASD, whereas Duphaston use during pregnancy, the longer interpregnancy interval and birth height were associated with decreased odds of ASD. These findings are pertinent as many identified factors may be preventable or modifiable, underscoring the importance of timely and appropriate public health strategies aimed at disease prevention in pregnant women to reduce ASD incidence.

## Introduction

Autism spectrum disorder is a neurodevelopmental disorder by the *Diagnostic and Statistical Manual of Mental Disorders,* the *5th Edition* (DSM-5). It is identified by limited repeating patterns of behavior, activities, and interests, as well as impaired social interaction and communication^1^. A systematic review of research articles spanning from 2012 to 2021 indicates that the worldwide median prevalence of ASD in children stands at 1%^2^. Nevertheless, this reported percentage may not fully capture the actual prevalence of ASD in low- and middle-income nations, potentially leading to underestimations. In 2016, data compiled by the CDC's Autism and Developmental Disabilities Monitoring (ADDM) Network revealed that approximately one out of 54 children in the United States (one out of 34 boys and one out of 144 girls) received a diagnosis of ASD. This marks a ten percent increase from the reported rate of one out of 59 in 2014, a 105 percent increase from one out of 110 in 2006, and a 176 percent increase from one out of 150 in 2000^3^. According to the most recent update from the CDC’s ADDM Network, one out of 36 (2.8%) 8-year-old children has been diagnosed with ASD. These latest statistics exceed the 2018 findings, which indicated a rate of 1 in 44 (2.3%)^4^. To our understanding, there is no existing registry for ASD in the Republic of Armenia (RA). Additionally, there is no available data concerning the incidence and prevalence of ASD in the country.

The etiology of ASD remains unclear despite substantial research on the disorder; yet, important advances have been made in identifying some of the disorder's genetic and neurobiological underpinnings. It has been discovered that ASD is heritable, with environmental variables also being involved^5–7^. According to certain research, ASD is associated with both hereditary and environmental factors^5,8,9^. It is especially important to identify environmental risk factors because, unlike genetic risk factors, they can be prevented.

There are more than 20 pre-, peri- and neonatal risk factors associated with ASD^10–12^. Prenatal risk factors that have been associated with ASD involve parental age^13^, interpregnancy interval^14,15^, immune factors (such as autoimmune diseases, both viral and bacterial infections during pregnancy)^16,17^, medication use (especially antidepressants, anti-asthmatics, and anti-epileptics)^18–20^, maternal metabolic conditions (such as diabetes, gestational weight gain, and hypertension)^21–23^, and maternal dietary factors (such as folic acid and other supplement use, maternal iron (Fe) intake, as well as maternal vitamin D levels)^24–29^.

Numerous studies indicate that an increased risk of ASD is linked to several perinatal and neonatal factors. These factors include small gestational age or preterm birth, gestational small or large size, the use of labor and delivery drugs^30–33^. The risk of ASD associated with cesarean delivery is also a subject of continuous discussion^34–36^. Overall, there is no apparent link between assisted conception and a notably higher risk of ASD, however some particular therapies might make ASD more likely.

This study aimed to determine main pre-, peri- and neonatal risk factors linked to ASD among Armenian children. The following research questions were derived to address the objectives of the study:

- What are the primary prenatal risk factors associated with the development of ASD among Armenian children?
- How do perinatal factors such as maternal complications during childbirth, labor mode, labor interventions, use of labor-inducing drugs, contribute to the risk of ASD in Armenian children?
- What neonatal factors, such as birth weight and gestational age, are linked to the likelihood of ASD diagnosis among Armenian children?
- How do socio-demographic factors, such as parental education, gender of the child, number of kids in the family, sequence of the kid, influence the relationship between pre-, peri-, and neonatal risk factors and risk of ASD among Armenian children?

To the best of our knowledge, this was the first study of its kind conducted in Armenia that focused on a variety of factors linked to ASD.

## Results

The analysis encompassed a total sample of 497 participants, consisting of 168 children diagnosed with ASD and 329 children without ASD. The descriptive analysis revealed significant differences between the cases and controls on several socio demographic variables as well as prenatal, peri- and neonatal risk factors (see Tables 1, 2, 3 and 4).

**Table 1:** Socio demographic characteristics of participants.

Variable	ASD cases (%)	Controls (%)	*p*-value
*Gender*			
Male	82.14	52.89	< 0.01
Female	17.86	47.11	
*Number of kids in family*			
One child	29.94	17.02	< 0.01
Two children	53.89	53.19	
More than two children	16.17	29.79	
*Sequence of the child*			
First	67.86	49.54	< 0.01
Second	26.19	36.00	
Third and more	5.95	14.46	
*Parents’ Marital Status*			
Married	89.76	95.72	< 0.05
Other (single, widowed, divorced)	10.24	4.28	
*Paternal Education*			
University degree	61.90	74.46	< 0.05
Other	38.10	25.54	
*Maternal Education*			
University degree	68.45	77.44	< 0.05
Other	31.55	22.56	

Data presented as mean ± standard deviation (SD) for continuous variables and percentages for categorical variables. *p* < 0.05 was considered statistically significant for this study.

**Table 2:** The summary of prenatal risk factors.

Variable	Cases	Controls	*p*-value
*Paternal age*	31.5 ± 6.0	31.5 ± 5.9	> 0.05
*Maternal age*	27.9 ± 4.9	27.6 ± 5.1	> 0.05
*Interpregnancy interval (in months)*	12.9 ± 26.6	23.7 ± 36.1	< 0.01
*Pregnancy process*			
Without complications	57.14%	91.46%	< 0.01
With complications	42.86%	8.54%	
*Pregnancy sequence*			
First	61.31%	46.77%	< 0.01
Second	23.21%	30.46%	
Third and other	15.48%	22.77%	
*Miscarriage before pregnancy*			
No	86.23%	86.28%	> 0.05
Yes	13.77%	13.72%	
*Abortion before pregnancy*			
No	91.07%	87.46%	> 0.05
Yes	8.93%	12.54%	
*Infectious diseases during pregnancy*			
No	70.24%	88.72%	< 0.01
Yes	29.76%	11.28%	
*Other Diseases during pregnancy*			
No	79.52%	88.11%	< 0.05
Yes	20.48%	11.89%	
*Anemia during pregnancy*			
No	82.04%	88.72%	< 0.05
Yes	17.96%	11.28%	
*Stress during pregnancy*			
No	43.98%	89.02%	< 0.01
Yes	56.02%	10.98%	
*Stress during pregnancy by trimesters*			
No stress	47.06%	91.25%	< 0.01
1^st^ trimester	14.38%	0.94%	
2^nd^ trimester	11.11%	4.06%	
3^rd^ trimester	8.50%	1.88%	
Whole pregnancy	18.95%	1.88%	
*Medication use during pregnancy*			
No	58.33%	82.26%	< 0.01
Yes	41.67%	17.74%	
*Other medication use*			
No	36.9%	79.64%	< 0.01
Yes	63.1%	20.36%	
*Supplement use during pregnancy*			
No	39.63%	35.06%	> 0.05
Yes	60.37%	64.94%	
*Vit D level during pregnancy*			
Normal	12.05%	46.95%	< 0.01
Not normal	5.42%	2.13%	
Don’t know	82.53%	50.91%	
*Maternal weight gain*	15.4 ± 8.7	13.9 ± 5.4	< 0.05

Data presented as mean ± SD for continuous variables and percentages for categorical variables. *p* < 0.05 was considered statistically significant for this study.

**Table 3:** Other medication use specified.

Variable	Cases (%)	Controls (%)	*p*-value
*Vitamins*			
No	87.5	96.05	< 0.01
Yes	12.5	3.95	
*Anticoagulants*			
No	92.86	98.78	< 0.01
Yes	7.14	1.22	
*Paracetamol*			
No	95.24	98.48	< 0.05
Yes	4.76	1.52	
*MgB6*			
No	76.79	94.53	< 0.01
Yes	23.21	5.47	
*Duphaston*			
No	88.69	95.14	< 0.01
Yes	11.31	4.86	
*Iron preparation*			
No	92.86	97.57	< 0.05
Yes	7.14	2.43	
*No-spa*			
No	98.21	100	< 0.05
Yes	1.79	0	
*Calcium preparation*			
No	96.43	99.39	< 0.05
Yes	3.57	0.61	
*Antibiotics*			
No	94.64	99.39	< 0.01
Yes	5.36	0.61	
*Utrogestan*			
No	97.62	99.7	< 0.05
Yes	2.38	0.3	

Data presented as mean ± SD for continuous variables and percentages for categorical variables. *p* < 0.05 was considered statistically significant for this study.

**Table 4:** The summary of perinatal and neonatal risk factors.

Variable	Cases	Controls	*p*-value
*Gestational age*			
37–42 weeks	83.33%	91.77%	< 0.05
Less or high than 37–42 weeks	16.67%	8.23%	
*Birth weight (in grams)*	3137.8 ± 523.5	3176.9 ± 391.8	> 0.05
*Birth height*	50.4 ± 2.6	50.9 ± 2.5	< 0.05
*Labor mode*			
Vaginal delivery	70.83%	77.74%	> 0.05
C-section	29.17%	22.26%	
*Labor interventions*			
No	60.24%	82.77%	< 0.01
Yes	39.76%	17.23%	
*Labor-inducing drugs*			
No	60.24%	78.96%	< 0.01
Yes	39.76%	21.04%	

Data presented as mean ± SD for continuous variables and percentages for categorical variables. *p* < 0.05 was considered statistically significant for this study.

The summary of socio demographic characteristics (Table 1). Among the cases (the ASD group), the distribution of gender of the child was significantly different to that in the control group. More specifically, while the distribution of male and female were balanced in the control group (52.89% and 47.11% respectively), the proportion of male children was significantly higher in the ASD group (82.14% and 17.86% respectively, *p* < 0.01). Furthermore, the number of children in the families of cases and controls were slightly different. While the proportion of cases and controls who had two children were similar, families with one child were slightly higher in the ASD group compared to the control group (29.94% and 17.02% respectively, *p* < 0.01). This picture is reversed with respect to the number of families with more than two children (16.17% and 29.79% respectively). A higher percentage of ASD cases are the first child in the family compared to controls (67.86% vs. 49.54%, *p* < 0.01). The proportion of non-married families (those that reported to be single, widowed, divorced etc.) were higher in the ASD group compared to the control group (10.24% and 4.28% respectively, *p* < 0.05). The distribution of the level of educational attainment of the parents were also different between the groups. More specifically, the prevalence of university degree among the cases were somewhat lower compared to that in the control group.

The summary of prenatal risk factors (Table 2). With respect to prenatal risk factors, there were significant differences between the cases and controls in interpregnancy intervals, self-reported complications and diseases, medication use, vitamin D levels, maternal weight gain, and the self-reported stress during pregnancy. More specifically, the cases had on average lower interpregnancy intervals compared to the controls (M = 12.9 and M = 23.7 months respectively, *p* < 0.01). The cases more frequently reported to have had complications during the pregnancy compared to the controls (42.86% and 8.54% respectively, *p* < 0.01). The prevalence of reported infectious diseases, other diseases and anemia during the pregnancy were also somewhat higher among the cases compared to the control group. The use of medications was higher among the cases compared to the control group (41.67% and 17.74% respectively, *p* < 0.01). Various medications including vitamins, anticoagulants, Paracetamol, MgB6, Duphaston, iron preparation, No-spa, calcium preparation, antibiotics, and Utrogestan showed significant differences in usage between cases and controls (all *p* < 0.05) (Table 3). The maternal weight gain among the cases was on average higher among the cases compared to the control group (M = 15.4 and M = 13.9 kg respectively, *p* < 0.05). The self-reported stress was also more frequent among the cases compared to the controls (56.02 and 10.98% respectively, *p* < 0.01). Specifically, comparing data on self-reported stress during different pregnancy periods, it was obvious that 47.06% of mothers of cases and 91.25% of mothers in the control group reported no stress experienced during pregnancy. During the first trimester, 14.38% of mothers with cases of autism reported stress, whereas only 0.94% of mothers in the control group reported stress. In the second trimester, 11.11% of mothers with cases of autism reported stress, compared to 4.06% of mothers in the control group. During the third trimester, 8.50% of mothers with cases of autism reported stress, while 1.88% of mothers in the control group reported stress. Across the entire pregnancy, 18.95% of mothers with cases of autism reported stress, compared to 1.88% of mothers in the control group. The differences in stress levels between the two groups were statistically significant, indicating a potential link between maternal stress during pregnancy and the odds of autism spectrum disorder in offspring.

The summary of perinatal and neonatal risk factors (Table 4). The interpretation of the data comparing various peri- and neonatal risk factors between cases (individuals with ASD) and controls (individuals without ASD) are shown below. 83.33% of cases and 91.77% of controls were born within 37–42 weeks of gestation, with a statistically significant difference (*p* < 0.05), whereas 16.67% of cases and 8.23% of controls were born either preterm (before 37 weeks) or post-term (after 42 weeks), also showing a significant difference. There was no statistically significant difference in birth weight between cases and controls (M = 3137.8 and M = 3176.9 g respectively, *p* > 0.05). The mean birth height was slightly lower for cases compared to controls (M = 50.4 and M = 50.9 cm), with a statistically significant difference (*p* < 0.05). No statistically significant difference was reported regarding mode of labor. According to the data interventions during labor were reported more in ASD group compared to controls (39.76% and 17.23% respectively, *p* < 0.01). Also, labor-inducing drugs were administered more in cases compared to the controls (39.76% and 21.04%, *p* < 0.01).

### The results of multivariable logistic regression

The multivariable logistic regression analysis indicated significant associations between sociodemographic, prenatal, perinatal and neonatal risk factors. More specifically, male children have 4 times higher odds of having ASD compared to female children (OR = 4.21, CI 2.33–7.63). Among prenatal factors, the maternal weight gain, use of MgB6, the self-reported stress during the pregnancy, as well as pregnancy with complications were associated with a significant increase in the odds of ASD, whereas use of Duphaston was associated with decreased odds of ASD (see Table 5). Additionally, the longer interpregnancy interval was associated with decreased odds of ASD diagnosis (OR = 0.708, CI 0.52–0.97). Among peri- and neonatal factors, use of labor-inducing drugs was associated with increase in the odds of ASD diagnosis (OR = 2.295, CI 1.3–4.1), while birth height showed association with decrease in odds (OR = 0.788, CI 0.6–1.0).

**Table 5:** Results of Multivariable Logistic regression.

Variable	OR	*p*-value	2.5%	97.5%
Male gender	4.211	0.000	2.325	7.626
Interpregnancy Interval	0.708	0.033	0.515	0.972
Maternal weight gain	1.203	0.171	0.924	1.566
MgB6	5.712	0.000	2.335	13.973
Duphaston	0.239	0.036	0.063	0.910
Stress during 1^st^ trimester	10.615	0.001	2.765	40.757
Stress during 2nd trimester	2.253	0.118	0.813	6.245
Stress during 3rd trimester	6.710	0.003	1.908	23.601
Stress during whole pregnancy	9.818	0.000	2.941	32.772
Labor-inducing drugs	2.295	0.005	1.294	4.071
Birth height	0.788	0.094	0.596	1.041
Pregnancy process with complications	5.485	0.000	2.753	10.926

## Discussion

Our study provides comprehensive insights into the multifaceted nature of ASD, elucidating the intricate relationships between sociodemographic, prenatal, perinatal, and neonatal factors and ASD risk.

Our findings highlight significant gender disparities in ASD prevalence, with a notably higher proportion (4:1) of male children in the ASD group. This aligns with existing literature demonstrating a male predominance in ASD diagnosis^37^. Meanwhile, Loomes et al. reported 3:1 male-to-female ratio referring to a diagnostic gender bias, where girls meeting the criteria for ASD are at an elevated risk of not receiving a clinical diagnosis^38^. Furthermore, our study highlights the potential impact of family structure on the likelihood of ASD occurrence, indicating higher ASD rates among first-born children and in households where the parents are non-married (divorced, widowed, separated, etc.). A study conducted by Ugur et al. yielded comparable findings, suggesting that the prevalence of being the eldest child was higher in the ASD group compared to the control group^39^. Contrary to this, research conducted in the United States found no evidence to suggest that children diagnosed with ASD are more likely to live in households not composed of both their biological or adoptive parents compared to children without ASD^40^.

The association between prenatal risk factors and ASD risk underscores the importance of maternal health during pregnancy. Our findings suggest that factors such as lower IPIs, maternal complications and diseases during pregnancy, medication use, vitamin D levels, maternal weight gain and maternal self-reported stress during pregnancy may increase the odds of ASD in offspring. It is crucial to note that the higher number of firstborn children among cases compared to the control group could introduce bias when accurately estimating the association between IPI and ASD. Therefore, the coefficients of IPI should be interpreted cautiously. Despite this, we opted not to remove this variable from the model, as IPI is recognized as an important factor in existing literature. Several studies report different results regarding long and short IPIs and ASD risk^14,15,41,42^. The underlying reasons for the link between ASD and short and long IPIs may differ. Short IPIs could be associated with maternal nutrient depletion, stress, infertility, and inflammation, whereas long IPIs may be linked to infertility and related complications. According to our results the frequency of self-reported complications during pregnancy was notably higher among children with ASD compared to the controls. Additionally, there was a somewhat higher prevalence of reported infectious diseases, other illnesses, and anemia during pregnancy among the cases compared to the control group. Several previous investigations have associated maternal hospitalization resulting from infection during pregnancy with an elevated risk of ASD. This includes a substantial study involving over two million individuals, which indicated an increased risk associated with viral and bacterial infections during the prenatal period^12,17^. Furthermore, our study results indicate that medication usage during pregnancy was more common among the mothers of cases than the controls. Notably, various medications, including vitamins, anticoagulants, Paracetamol, MgB6, Duphaston, iron preparation, No-spa, calcium preparation, antibiotics, and Utrogestan (micronized progesterone), exhibited significant differences in usage between the cases and controls. According to the results of multiple logistic regression analysis use of MgB6 was associated with a significant increase in the odds of ASD, whereas use of Duphaston (Dydrogesterone), a progestin medication, was associated with decreased odds of ASD. Emphasizing the potential impact of additional variables in evaluating the link between Duphaston and ASD is essential. There is a possibility of factors overlooked in our study. However, after analyzing the included variables, no significant confounding effects were detected. This assessment involved scrutinizing the Cramer’s V value between Duphaston and other variables, and sequentially introducing new variables into the model to evaluate changes in the coefficients of Duphaston. In both cases, no significant confounding effects emerged. In contrast to our results certain researchers have shown that the use of supplements during pregnancy is linked to a decreased risk of ASD in offsprings compared to those whose mothers did not take supplements during pregnancy^43,44^. The results of an epidemiology study conducted by Li et al. have showed that prenatal progestin exposure was strongly associated with ASD prevalence, and the experiments in rats showed that prenatal consumption of progestin-contaminated seafood induced autism-like behavior^45^. On the other hand other authors suggest that insufficient maternal progesterone levels might contribute to both obstetrical complications and ASD development^46^. The observed association regarding MgB6 use could potentially be influenced by an unmeasured confounding variable in our study. This warrants further investigation and consideration in future research. Additionally, our study highlighted another modifiable risk factor for ASD that was significantly associated with higher odds of ASD: maternal gestational weight gain. This factor retained its significance even in the multivariable analysis. This finding is consistent with the results of several studies which have shown that maternal metabolic conditions like diabetes, gestational weight gain, and hypertension have been associated with mechanisms pertinent to ASD, such as oxidative stress, fetal hypoxia, and chronic inflammation^23,47^. These conditions can induce prolonged or acute hypoxia in the fetus, which might pose a substantial risk factor for neurodevelopmental disturbances.

Furthermore, our findings suggest that the self-reported stress during pregnancy was associated with a significant increase in the odds of ASD. When comparing self-reported stress levels during different pregnancy periods, a notable disparity emerged. The statistically significant differences in stress levels between the two groups were reported in all trimesters of pregnancy suggesting a potential correlation between maternal stress during pregnancy and the odds of autism spectrum disorder in offspring. Several authors report comparable findings suggesting that prenatal maternal stress show significant association with both autistic traits and Attention Deficit Hyperactivity Disorder (ADHD) behaviors^48–51^. Various mechanisms could be suggested to explain the link between prenatal stress and likelihood of ASD. For instance, stress during pregnancy can trigger physiological changes in the mother's body, such as increased cortisol levels and alterations in immune function, which may impact fetal development and contribute to the risk of ASD. Also, stress may affect placental function, leading to adverse changes in the transfer of nutrients and oxygen to the fetus. Furthermore, prenatal stress can influence gene expression in both the mother and the developing fetus. Certain genes involved in brain development and the stress response system may be affected, potentially increasing the risk of ASD. Additionally, maternal stress may influence parenting behaviors and interactions with the child after birth. High levels of maternal stress may affect the quality of caregiving, which in turn can impact the child's social and emotional development, potentially contributing to ASD risk. Lastly, stress during pregnancy could induce epigenetic modifications, which are alterations in gene expression that occur without changes in DNA sequence. These modifications might affect neurodevelopmental processes, making individuals more susceptible to ASD.

Our study reports notable statistical difference among cases and controls regarding gestational age. According to the results 16.67% of cases were born either preterm (before 37 weeks) or post-term (after 42 weeks), highlighting another significant distinction. Early gestational age is linked with unfavorable health consequences, such as developmental delays and subsequent intellectual impairments throughout childhood and adolescence. Similar results are reported by several authors as well^30,31,52^. According to our study results birth height was associated with decrease in odds of ASD, however there was no statistically significant difference in birth weight between cases and controls. Some authors demonstrated that infants with birth weights of < 2.5 kg were associated with ADHD and ASD^53,54^.

Other than the previously mentioned factors the results of our study have demonstrated that use of labor-inducing drugs was associated with increase in the odds of ASD. The study participants did not report or specify the type of used labor-inducing drugs. Recent investigations have indicated a potential correlation between the utilization of drugs during labor and delivery and the emergence of ASD^55,56^, especially given the increased usage of epidurals and labor-inducing medications in the past 30 years. However, conflicting findings exist, with some studies suggesting no link between the administration of labor-inducing drugs and the risk of ASD development^57,58^. Recent findings from Qiu et al. propose a potential link between maternal labor epidural analgesia and the risk of ASD in children, particularly when oxytocin was concurrently administered. However, oxytocin exposure in the absence of labor epidural analgesia did not show an association with ASD risk in children^59^. The potential link between the use of labor-inducing drugs and the risk of ASD is complex and not yet fully understood. However, several mechanisms have been proposed to explain this association: for example, labor-inducing drugs, such as oxytocin and prostaglandins, can affect hormonal levels in both the mother and the fetus. These hormonal changes may impact brain development and neural connectivity, potentially increasing the risk of ASD. In addition, labor induction may increase the risk of oxygen deprivation (hypoxia) during labor. Prolonged hypoxia during birth has been linked to adverse neurological outcomes, including an increased risk of neurodevelopmental disorders like ASD. Furthermore, labor induction can lead to an inflammatory response in both the mother and the fetus. This immune system activation may affect neurodevelopmental processes and contribute to the development of ASD. Overall, the relationship between labor-inducing drugs and ASD risk is multifactorial and likely involves interactions between genetic, environmental, and biological factors. Further research is needed to elucidate the specific mechanisms underlying this association.

To our knowledge this is the first study identifying potential pre-, peri- and neonatal risk factors associated with ASD in Armenia.

## Limitations and strength

While our study possesses a retrospective design, a notable limitation, it depended on parental recall for details dating back several years. Additionally, the sample was not balanced in terms of gender, with more male children included, potentially introducing selection bias. Despite the relatively small sample size multivariable analysis using the presence or absence of ASD as the dependent variable was implemented to address potential confounding factors.

Nevertheless, our study included a representative sample of the Armenian ASD population, evaluating various factors in comparison with a randomly selected control group matched for age. All ASD diagnoses were made according to DSM-5 by professionals (psychiatrists, pediatricians, neurologists, speech therapists and developmental psychologists), and face-to-face interviews with parents at the time of the interview minimized the risk of information bias.

## Conclusion

**Figure 1:**
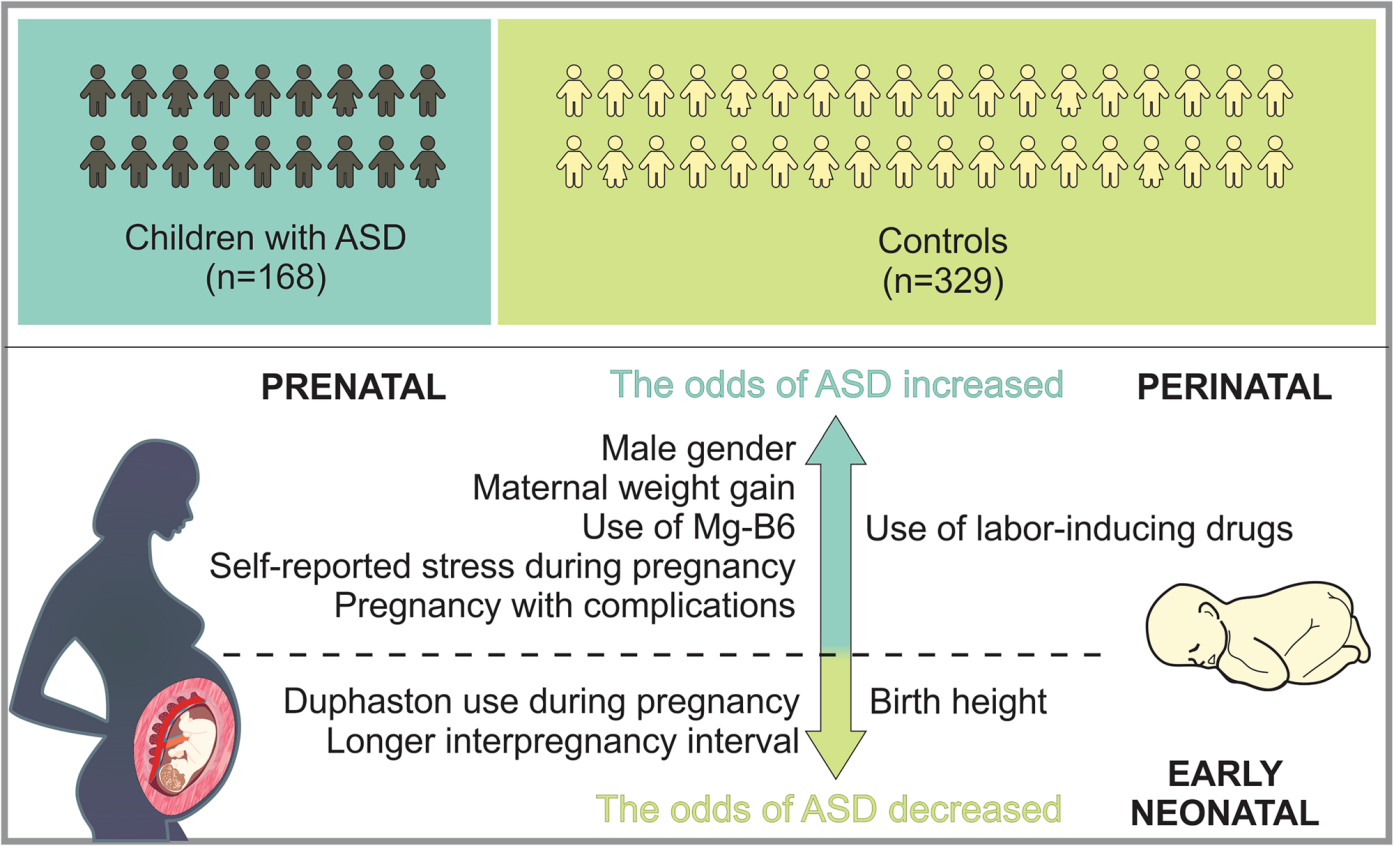
Sum up scheme showing prenatal, perinatal and neonatal factors which increase, as well as decrease the odds of ASD.

Our findings indicated that male gender, maternal weight gain, MgB6 usage, self-reported stress during pregnancy, pregnancy complications, and labor-inducing drugs were linked to a significant rise in ASD odds (Fig. 1). Conversely, the use of Duphaston during pregnancy, longer interpregnancy intervals, and higher birth height were associated with reduced odds of ASD (Fig. 1). These observations underpin the significance of regional investigations to uncover the unique environmental factors contributing to ASD. The implications are profound, as several identified factors may be preventable or adjustable, highlighting the urgency of implementing evidence-based practices and public health interventions. Emphasizing a culture of health promotion, screenings, timely diagnosis, and disease prevention strategies, particularly among pregnant women, holds promise for reducing ASD and related disorders. Moreover, further prospective and focused research is imperative to discern the interplay between various factors and gene-environment interactions that may serve as potential ASD risk factors. Enhanced understanding in this area could lead to earlier detection and improved ASD management. Future studies incorporating analyses of biological samples for genetic, epigenetic, and inflammatory markers will be pivotal in elucidating underlying mechanisms and ushering in a new phase of research focusing on modifiable risk factors for developmental disorders.

## Methods

### Subjects

The study population comprised of 497 participants, of which 168 were children with ASD and 329 were typical development controls. The subject recruitment was done during 2021 to 2022. The controls and children with ASD were age matched (3–18 years). The subjects were formally diagnosed with ASD according to DSM-5 by professionals (pediatricians, neurologists, speech therapists and developmental psychologists). The children with ASD were recruited at MY WAY Educational and Rehabilitation Center in Yerevan. Inclusion criteria for cases were age between 3 and 18 years with ASD with diagnosis confirmed using DSM-V. Exclusion criteria for cases were other neurodevelopment disorders other than ASD. The control participants were randomly selected in the same period at Muratsan Hospital Complex. They were not known to have any neurodevelopmental or behavioral disruptions that might be related to ASD. The control group consisted exclusively of individuals diagnosed with simple conditions like flu or a simple routine physical examination.

### Questionnaire and data collection

The self-reported questionnaire was completed via a face-to-face interview with the child’s parent. The questionnaire comprised three sections: various aspects of sociodemographic characteristics, prenatal risk factors, and perinatal/neonatal risk factors. Each section addressed specific questions related to these factors for comprehensive data collection. On average, the questionnaire was completed in about 15 to 20 min. The parent had the choice of accepting or refusing to complete the questionnaire. At the end of the process, the completed questionnaires were collected and sent for data entry by using SPSS 21 statistical software. The questionnaire was designed to acquire the information regarding risk factors of ASD. It consisted of different sections: sociodemographic characteristics (e.g., age, sex, family history of ASD, etc.), data on prenatal risk factors (e.g., pregnancy process, complications during pregnancy, infections during pregnancy, other diseases, stress, medication and supplement use during pregnancy, vitamin D level, etc.), questions related to maternal lifestyle risk factors (e.g., smoking, alcohol consumption, gestational weight gain). Respondents were also asked about peri- and neonatal risk factors, including gestational age, gestational size, the use of labor and delivery drugs, mode of delivery, etc.

### Ethics disclosure

The study protocol was approved by the Ethics Committee (N 8–2/20; 27.11.2020) of Yerevan State Medical University in line with the principles set forth in the Declaration of Helsinki. Written informed consent was obtained from all the parents of the participants prior to data collection.

### Statistical analysis

The data was processed and modelled in Python (version 3.11.6), an open- source software often used for data processing and modelling. The statistical analysis was performed by using the Statsmodels package (version 0.14.1)^60^.

Descriptive statistics were employed to summarize the characteristics of the study variables. Continuous variables were described using means and standard deviations, while categorical variables were presented as proportions. Prior to statistical analysis, numeric variables underwent standardization through the computation of z scores. To evaluate potential multicollinearity among predictor variables, the Variable Inflation Index (VIF) was computed. A predetermined threshold of 5 was established, and variables exceeding this threshold were considered indicative of multicollinearity. Bivariate analyses were conducted to explore associations between predictor variables and the outcome variable (presence or absence of autism diagnosis). T-tests were employed for continuous variables, while chi-squared tests were utilized for nominal and categorical variables. A multivariable logistic regression model was constructed to estimate the relationship between predictor variables and the outcome variable (autism diagnosis). Initially, a comprehensive strategy was employed by first fitting a null model and then iteratively introducing blocks of variables (e.g., socio demographic factors prenatal, peri- and neonatal factors). After introducing the new block of factors, a likelihood ratio test was conducted to evaluate the contribution of the added variables. If the *p*-value associated with the likelihood ratio test was insignificant (*p* > 0.05), the preference was given to a less complex model. In our analysis all added blocks had significant contribution to the overall fit of the model.

Subsequently, the significance of each variable was systematically assessed by applying a stepwise elimination technique whereby insignificant variables were progressively removed from the model. Similar to the above-mentioned process, at each step, a likelihood ratio test to evaluate the significance of the excluded variable was conducted. If the *p*-value associated with this test was found to be insignificant (*p* > 0.05), the adoption of a simpler model was favored. This iterative process allowed us to identify the most parsimonious model that retained statistically significant predictors while minimizing unnecessary complexity. Odds ratios (ORs) were computed, accompanied by 95% confidence intervals (CIs), to quantify the strength and direction of these associations.

## Data Availability

Data can be made available by the corresponding author upon reasonable request.
